# Boundaryless career and career success: the impact of emotional and social competencies

**DOI:** 10.3389/fpsyg.2015.01304

**Published:** 2015-09-01

**Authors:** Fabrizio Gerli, Sara Bonesso, Claudio Pizzi

**Affiliations:** ^1^Department of Management, Ca' Foscari University of VeniceVenice, Italy; ^2^Department of Economics, Ca' Foscari University of VeniceVenice, Italy

**Keywords:** emotional and social competencies, boundaryless career, career mobility, career success, career management

## Abstract

Even though, over the last two decades, the boundaryless career concept has stimulated a wide theoretical debate, scholars have recently claimed that research on the competencies that are necessary for managing a cross-boundary career is still incomplete. Similarly, the literature on emotional and social competencies has demonstrated how they predict work performance across industries and jobs but has neglected their influence in explaining the individual's mobility across boundaries and their impact on career success. This study aims to fill these gaps by examining the effects of emotional and social competencies on boundaryless career and on objective career success. By analyzing a sample of 142 managers over a period of 8 years, we found evidence that emotional competencies positively influence the propensity of an individual to undertake physical career mobility and that career advancements are related to the possession of social competencies and depend on the adoption of boundaryless career paths. This study also provides a contribution in terms of the evaluation of the emotional and social competencies demonstrated by an individual and of the operationalization of the measurement of boundaryless career paths, considering three facets of the physical mobility construct (organizational, industrial, and geographical boundaries).

## Introduction

Social, economic, and technological changes increasingly lead to a discontinuous and fragmented career context in which transitions occur more frequently than ever. As described by Feldman and Ng (2007), several factors have promoted individuals' career mobility over the last decade. Among them, the macroeconomic conditions have created geographic disparities in mobility opportunities and the perception of favorable economic conditions in some areas. Moreover, firms have downsized and outsourced to become more flexible, efficient, and innovative, changing career expectations from job security to continuous learning and to remain marketable in the external labor market. Social changes have led to increased upward mobility for female and minority employees, for instance through the implementation of diversity programs. Moreover, the extensive use of information and communication technologies has changed the way individuals interpret their job, promoting for instance virtual work that favors mobility and may decrease the sense of identification with the specific organization. Therefore, the traditional career model characterized by a full-time permanent job with a single employer has been progressively replaced by a contemporary career model which emphasizes inter-organizational mobility and temporary contracting arrangements (Sammarra et al., 2013). Recent research has provided empirical evidence of this shift toward career mobility in different countries. Kim (2013) showed that organizational mobility increased in the USA throughout the 1990s and early 2000s in a wide range of jobs. Lyons et al. (2015) provided evidence of organizational mobility across generations in Canada. The diffusion of boundaryless career concepts in the European context has been demonstrated by some studies (Chudzikowski et al., 2010). Bagdadli et al. (2003) analyzed the evolution of the Italian labor market and found that inter-organizational mobility is more frequent than in the past, “especially for younger generations, people working in professional occupations and new sectors, or those living in large northern towns” (2003, p. 796). Even though workers' mobility in the European Union remains modest compared to the United States, the number of mobile workers has increased sharply in absolute terms over the last decade (European Commission, 2014).

Thus, in the current labor market “individuals are, or should be, increasingly mobile and self-directed in their careers” (Gubler et al., 2014b, p. 23). In this regard, the notion of a boundaryless career (Arthur, 1994; Arthur and Rousseau, 1996) is becoming central for the understanding of contemporary career paths.

Even though, over the last two decades, the boundaryless career concept has stimulated a wide theoretical debate, its ability to adequately capture the nature of contemporary careers has been questioned (Rodrigues and Guest, 2014). Specifically, among the main critics, scholars argue that there has been a limited advancement in boundaryless career conceptualization, on the one hand, and a scantly empirical investigation of its determinants and outcomes, on the other.

Regarding the first point, as suggested by Inkson et al. (2012), it remains ambiguous which particular boundaries are crossed. Literature has ascribed primacy to the role of organizational boundaries, but this definition is rather limiting since it neglects other relevant domains of physical mobility, such as occupation, industry, and geography (Arthur and Rousseau, 1996; Sullivan and Arthur, 2006). In addition to these physical movements, boundaryless careers also involve a psychological dimension, namely the willingness “to initiate and pursue work-related relationships across organizational boundaries” (Briscoe et al., 2006, p. 31), also defined by Sullivan and Arthur as “the perception of the capacity to make transitions” (2006, p. 21). Thus, extant contributions seem not to take into account Arthur and Rousseau's (1996) initially much broader notion of this concept.

With regards to the second point, as recently claimed by Rodrigues and Guest (2014) “a theory of career boundaries needs to explain the antecedents and outcomes of preference (or perception) of boundary characteristics.” Since employees, in this contemporary career concept, are primarily responsible for managing their career path, individual agency (Gubler et al., 2014b) and personal attributes represent critical factors for career mobility. Literature has provided evidence of the impact of individual factors on actors' propensity to move across boundaries by considering their gender, level of education, age, and prior working experience. In this regard, prior studies provide evidence of a dual direction of physical career mobility for women that seems to depend on their relationships and commitments to others (Valcour and Tolbert, 2003; Mainiero and Sullivan, 2005; Forret et al., 2010). Indeed, women may undertake a cross-boundary career in order to meet husbands' career mobility or, on the other hand, they may turn down job offers which require family relocation or increased travel with negative consequences in terms of reduced time spent with their family.

In another study, Segers et al. (2008) explored the role of gender, age, education, and managerial experience in work motives associated with physical and psychological career mobility. The authors found that women score higher on motivation related to psychological mobility, while men present a higher score on motivation related to physical mobility. Moreover, younger people show a higher physical career mobility than older workers and a higher managerial experience or a higher education turned out to be positively related to motivators linked both to physical and psychological mobility. Chan et al. (2012) also showed that individuals whose careers are driven by entrepreneurial and leadership motivations present higher boundaryless career attitudes, while those primarily motivated to pursue professional careers express more traditional career attitudes. Finally, a more recent study has considered proactive personality as an explanatory factor of boundaryless career attitudes (Uy et al., 2015).

Besides these predictors, boundaryless career literature has contributed to theoretically defining, in three main dimensions, the competencies that individuals require in order to undertake successful career transitions, namely: “knowing why,” associated with an individual's motivational energy to understand oneself, explore different possibilities, and adapt to constantly changing work situations; “knowing whom,” which relates to the career-related networks, mentoring, and contacts of an individual both inside and outside the organization; and “knowing how,” which involves career identity and job-related knowledge which accumulates over time (DeFillippi and Arthur, 1994; Arthur et al., 1995; Eby et al., 2003). Apart from the professional knowledge required to perform a specific job, these studies have drawn attention to the role of competencies as antecedents of a boundaryless career and have underlined the importance of both personal and interpersonal skill dimensions (Akkermans et al., 2013). However, scholars have recently claimed that research on the competencies that are necessary for managing a cross-boundary career is still incomplete, and the dimensions developed and measured in prior studies are inconsistent (Wang, 2013). Moreover, the conceptualization of the three career competencies (knowing-why, knowing-how, and knowing-whom) is limited because it only considers the “repositories of knowledge” individuals may acquire and use throughout their professional experiences, especially when moving across boundaries. This stream of research neglects the behavioral dimension of individual competency that goes beyond the mere knowledge and focuses on the intent and the related actual behaviors that enable individuals to pursue superior results in their career path. In this regard, the field of study that has analyzed those individual competencies required for making successful career transitions may benefit from the well-established competency-based literature that has conceptualized over the last decades the emotional and social competencies that distinguish individuals who achieve outstanding performance (McClelland, 1973; Boyatzis, 1982, 2009; Spencer and Spencer, 1993).

Furthermore, the literature claims that transitions to different organizations are motivated by the need of individuals to seek opportunities that maximize their extrinsic rewards, in terms of a higher level of objective career success, leading to an increase in the salary level, a better position on the hierarchical ladder or a better contract (Mao, 2004; Cheramie et al., 2007). However, empirical support for this claim is still limited.

Similarly, the literature on emotional and social competencies (ESCs) has demonstrated how they predict job performance across industries and jobs (Boyatzis, 1982, 2009; Spencer and Spencer, 1993; Goleman, 1998; Williams, 2008; Beigi and Shirmohammadi, 2011; Zhang and Fan, 2013), but has neglected their influence in explaining the individual's mobility across boundaries and, in turn, to attain career success.

This study aims to contribute to fill these gaps by examining the relationship between ESCs, on the one side, and physical career mobility and career success, on the other. Specifically, we address the following research question: Do ESCs spur individuals to cross boundaries in their career path and to pursue a higher objective career success over time?

This paper adds to career management and competency-based literature in several ways. First, it contributes to building the conceptual bridge between the literature on the boundaryless career and the research stream on ESCs, by considering them as antecedents of physical career mobility across organizational, industrial, and geographical boundaries. In so doing, we also address the need of extending the conceptualization of physical mobility beyond the mere organizational boundaries dimension. Secondly, it adds to the debate on ESCs and boundaryless career by investigating their effects on objective career success. Thirdly, it provides a dynamic perspective in studying the impact of ESCs on the individual's career mobility and success over time. Indeed, contrary to prior research in which data were primarily collected at only one point in time, we adopt a time-lagged design to investigate how ESCs influence the evolution of managers' careers.

This paper is organized as follows. In the next section, we propose a framework that aims to bridge career management and the competency-based literatures, introducing the research hypotheses on the relationship between the possession of ESCs, the pursuit of a physical boundaryless career, and the attainment of a higher career success. Next, in the Methods Section we illustrate the setting and the time-lagged design adopted in this study. Afterwards, we present the results of the empirical analysis, and in the final section we discuss the implications and future research avenues in terms of career development.

## Theoretical background and research hypotheses

### Emotional and social competencies as predictors of a boundaryless career

The literature on boundaryless careers has theoretically advocated that individuals, in order to cope with the complexity of the current career environment, need to acquire “three ways of knowing” (knowing-why, knowing-how, and knowing-whom), to successfully navigate their boundaryless careers (DeFillippi and Arthur, 1996; Eby et al., 2003; Colakoglu, 2011). As recently highlighted (Fleisher et al., 2014), these career competencies are acquired through the sequences of individuals' work experiences, and are conceived in terms of information, knowledge, and relationships individuals may deploy in their career path. However, recent research has demonstrated that in order to successfully perform in their careers, individuals require not just knowledge about their motivation and identity, skills, and inter- and intra-organizational networks, but also a set of behavioral abilities, namely emotional and social competencies, that enable them to pursue effectiveness (Brown et al., 2003; Emmerling and Cherniss, 2003; Williams, 2008; Beigi and Shirmohammadi, 2011; Emmerling and Boyatzis, 2012; Zhang and Fan, 2013).

The competency framework is based on the premise that individuals' past behaviors predict their future outcome (Spencer and Spencer, 1993; Boyatzis, 2009). Originally introduced by McClelland (1973) and then later refined by Boyatzis (1982) and Spencer and Spencer (1993), the notion of behavioral competency is defined as “the underlying characteristics of a person that lead to or cause effective or superior performance” (Boyatzis, 1982) and has been recently described as “a set of related but different sets of behaviors organized around an underlying construct called intent” (Boyatzis, 2009). According to these definitions, a competency is manifested by a set of behaviors that are driven by motives and are instrumental in attaining work outcomes (Boyatzis and Kelner, 2010).

Prior studies that aimed to identify those behavioral competencies that distinguish outstanding from average performers across professions and sectors (Boyatzis, 1982, 2009; Spencer and Spencer, 1993; Emmerling and Boyatzis, 2012), have discriminated between emotional and social competencies. The former refers to the ability of recognizing, understanding, and managing emotional information about oneself, for example planning, achievement orientation, adaptability, self-control, whereas social competencies concern the ability of recognizing, understanding, and managing emotional information about others, such as empathy, teamwork, negotiation, networking, conflict management, and leadership (Goleman, 1995; Boyatzis, 2009).

Prior research has shown the impact of emotional and social competencies on work performance across a wide range of roles, in terms, for example, of financial performance (McClelland, 1998; Boyatzis, 2006), service quality (Beigi and Shirmohammadi, 2011), project performance (Zhang and Fan, 2013), customer satisfaction (Williams, 2008), and effectiveness in public service (Sharma, 2013). However, to date ESCs have not been investigated as predictors of career decisions with specific regard to boundaryless career paths.

Addressing this issue we aim to understand if ESCs are antecedents of actual career moves, namely physical mobility. In this study, we do not investigate the second dimension of boundaryless career, that is psychological mobility, since it only captures the individual perception or belief about the capacity to move across boundaries and it does not imply an actual shift toward new job opportunities (Briscoe et al., 2006; Sullivan and Arthur, 2006). Indeed, as recently demonstrated by Vansteenkiste et al. (2013), the willingness to envision a variety of career options does not necessarily enhance a jobseeker's search success. Moreover, this subjective dimension of boundaryless career may be stimulated by self-reflection and awareness about the personal resources that individuals perceive to possess, that are only one component of emotional competencies. Since emotional and social competencies are manifested by a set of actual behaviors that spur individuals toward the attainment of superior performance, we aim to understand their impact on objective career changes.

Critics of the boundaryless career concept have highlighted the possible downsides individuals face when they undertake physical mobility. Crossing career boundaries may create uncertainty and generate stress (Rodrigues and Guest, 2010) related to the job insecurity that individuals face when they perceive threats to the continuity of one's employability and to the uncertainty regarding the quality of the subsequent employment (Colakoglu, 2011). This perception of ambiguity may increase if the individuals not only change employer but if they also face a new industrial context and operate in a different geographical location. For these reasons, we expect that individuals who demonstrate emotional competencies, and are thus better able to understand their feelings and are more successful in regulating them through a thorough awareness of their self, may obtain a higher efficacy in planning and managing career-related issues, and in turn be more proactive and prone to identifying career opportunities. Specifically, individuals who possess a higher level of emotional competencies are expected to be better able to envisage and organize their future, setting desired career goals, defining a series of actions toward the achievement of their goals, moderating risks in a situation prior to taking action, and anticipating obstacles. Moreover, the possession of emotional competencies allows the individual to thrive even in adversity (Sharma, 2011), remain calm in stressful situations/settings and to easily adapt to changing circumstances, competencies often associated with a tolerance for ambiguity and uncertainty. Thus, we expect that:

*Hypothesis 1*. Emotional competencies positively relate to a boundaryless career.

Another drawback usually ascribed to frequent career mobility is related to the limited propensity of these individuals to invest in their relationships at work or in their internal career development, due to their strong focus on frequent job changes and consequent limited time in the same position (Verbruggen, 2012). The incapacity to build and use contacts may negatively influence the recognition of opportunities for job mobility, and prevent individuals attaining subsequent career outcomes. Individuals pursuing physical boundaryless careers may require social competencies in order to develop and utilize networks of contacts that provide them with access to critical information and opportunities for attaining career mobility. Developing relationships with different professional communities with disconnected contacts (Burt, 1992) and nurturing them beyond the current employing organization provides not only information but also other important resources such as influence, reputation, and guidance, offering a competitive advantage to the individual. Besides, networking activities and social competencies may support individuals in better understanding different perspectives and settings through an empathetic listening and in influencing others in the effort of gaining continuous employability. Therefore, we maintain that:

*Hypothesis 2*. Social competencies positively relate to a boundaryless career.

### Emotional and social competencies, boundaryless career and career success

According to Arthur et al. (1989 p. 8) a career is an “evolving sequence of a person's work experiences over time,” and the accumulation of advancements arising from these work experiences is defined as career success (Judge et al., 1999). Career success has consistently been shown to favor people's well-being and organizational success (Pachulicz et al., 2008). It is conceptualized as “the positive psychological or work-related outcomes or achievements one accumulates as a result of work experiences” (Seibert et al., 1999, p. 417), and encompasses two dimensions: objective and subjective career success. The first has been conceived in terms of job progression and has been measured, for instance, by salary level and progression (Orpen, 1998; Wayne et al., 1999), number and speed of promotions earned (Judge et al., 1995; Seibert et al., 1999; Tharenou, 1999), and functional/managerial level or grade (Judge et al., 1995; Kirchmeyer, 1998; Tharenou, 1999), which serve as external perspectives for assessing progress. The second dimension, subjective career success, is associated with the concept of psychological success and primarily measured by variables such as career satisfaction and comparative judgements (Greenhaus et al., 1990; Judge et al., 1995; Heslin, 2003, 2005; Abele and Spurk, 2009).

Since in this study we are interested in the longitudinal impact of ESCs and boundaryless careers on objective advancements attained by individuals during their career path, we focus our attention on the objective career success rather than considering subjective perceptions in terms of feelings of accomplishment and satisfaction with one's career. Moreover, since the labor market and organizational changes have reduced the opportunities for only hierarchical advancements, nowadays objective career success includes both vertical and horizontal promotions. Therefore, we refer to career success as “any increases in level and/or any significant increases in job responsibilities or job scope” (Seibert et al., 2001, p. 227).

Despite the fact that competency-based research has produced consistent studies on the relationship between ESCs and job performance, only a few of them have devoted attention to the impact of emotional and social competencies on objective career success. These contributions have primarily focused only on single competencies that refer to the emotional and social competency constructs. Among them, Poon (2004) demonstrated that a person does not achieve objective career success (in terms of level of salary) simply because they are committed to their career, but a relevant role is played by the “emotion perception,” that is the ability to identify emotions in oneself and in others, which positively moderates the relationship between career commitment and objective career success. According to this study, emotional competencies enable individuals to better “assess their job skills and interests, set appropriate career objectives, develop realistic career plans, and obtain the developmental experiences needed to take advantage of career opportunities” (Poon, 2004, p. 377). Emotional competencies do not refer only to self-awareness but also to the abilities that enable an individual to manage his/her own emotions. For instance, prior research found that the individual's concern for working toward a standard of excellence and for developing challenging and specific goals, namely achievement orientation, enables individuals to improve their own performance and outperform others (McClelland, 1985). This positive impact on job performance may provide a higher visibility in the marketplace increasing the opportunity for getting career promotions (Kuijpers and Scheerens, 2006; Abele and Spurk, 2009). Also, the capacity to adapt to new working demands, to different groups and environments has been highlighted as a key competency in the career competency literature (DeFillippi and Arthur, 1994; Hall, 2004). Focusing on self-control, namely the effective regulation of thoughts, feelings, and behaviors (Converse et al., 2014), a recent study found a positive impact of self-control on salary, demonstrating that individuals who are more able to work under pressure and manifest stress tolerance are more willing and able to manage greater work-related responsibilities, and consequently more likely to access higher positions (Converse et al., 2012). Similarly, prior literature review has provided evidence on the impact of emotional competencies on job advancement (Zeidner et al., 2004). Therefore, we posit that:

*Hypothesis 3*. Emotional competencies positively relate to objective career success.

The ability to perceive and understand emotions in others, namely social competencies, may favor positive evaluations from others and supportive relationships that in turn impact on career advancements (Poon, 2004). Another study provided evidence on the significant and positive effect of the ability to build social relationships on salary growth (Wolff and Moser, 2009). Building and using contacts may yield a higher work performance, due to the possibility of relying on competent relations and to efficiently complete the tasks, and in turn increase performance ratings and salary. Moreover, as prior studies on social capital and career advancements have highlighted, access to information and resources increases reputation, and “the individual will be perceived as more powerful or influential in the organization” (Seibert et al., 2001, p. 224). Furthermore, the ability to nurture helping relationships through “coaching” activities not only increases the organizational and coachee's effectiveness, but also generates a positive emotional contagion from the coachee to the coach (Boyatzis et al., 2013), which can be positively evaluated for promotions to higher managerial roles. Thus, we expect that:

*Hypothesis 4*. Social competencies positively relate to objective career success.

Previous research has found contradictory evidence on the relationship between boundaryless career and objective career success. However, these studies have primarily considered salary attainment as a measure of career success and have relied on cross-sectional data (Verbruggen, 2012; Vinkenburg and Weber, 2012; Sammarra et al., 2013).

Crossing organizations, industries or geographical domains may stimulate individuals over time toward learning and professional development. Different and changing requirements in new jobs encourage individuals to continuously gain new education and training (Heilmann, 2011). According to Arthur and Rousseau (1996), individuals who undertake a boundaryless career possess a higher propensity to learn and to reflect on their strengths and weakness than individuals who embrace a traditional career. People who build experience in a variety of fields and roles strengthen their job profile, acquire a wider view of the phenomenon and consequently have more opportunities to be promoted. Indeed, for higher-level managerial roles, firms may prefer individuals with a generalist background rather than a specialist (Verbruggen, 2012). Moreover, as suggested by Feldman and Ng (2007), individuals who are not able to get promoted in the current organization, seek out opportunities for career advancement crossing organizational boundaries. Building upon these considerations we expect a positive effect of a boundaryless career on objective career success. Therefore, we suggest:

*Hypothesis 5*. A boundaryless career relates positively to objective career success.

## Methods

### Sample and procedure

We tested our hypotheses with data collected in a time-lagged study on a sample of Italian managers who participated in a 2-year part-time Executive MBA program from 2003 to 2012. At the end of each MBA edition (Time 1, “T1”) we collected data on the ESCs of its participants. In 2013 (Time 2, “T2”), we collected data on the overall career of the participants across the eight MBA cohorts at one point in time.

The sample used in this study was initially composed of 149 managers who responded to an online survey and for whom we had T1 and T2 data (34.5% of the total population of 432 participants in the MBA program). All participants to this study gave explicit consent to data collection for the purposes of this research, and all the researchers have been subject to the Ethical Code of their affiliating Institution.

To assess the non-response bias we compared some characteristics of the population with those of the managers who completed the survey in T2. The Chi-square test revealed no significant differences in terms of gender (*p* = 0.64), age at the beginning of the MBA (*p* = 0.67), and number of years of working experience (*p* = 0.58) between respondents and non-respondents. Due to the presence of missing values (four units) and outliers (three units), the final sample was reduced to 142.

In the sample, 122 respondents were males (86% of the whole sample) and the average age was 40.9 years at the end of the MBA (*S*.*D*. = 6.63). The average age of the males was 41.79 (*S*.*D*. = 6.38), while for females 35.35 (*S*.*D*. = 5.13). Concerning the sample's educational profile, 88.0% held a degree (87.0% of males and 97.0% of females). Considering the whole sample 47.2% held a degree in engineering, 22.4% in economics and management, 7.2% in law, 23.2% in other disciplines. As for their professional profile, on average the respondents had 14.6 years of working experience (*S*.*D*. = 5.81) and had been working for 7.7 years as managers (*S*.*D*. = 5.43). If we consider the sample's professional background according to the gender, females had 10 years of working experience (*S*.*D*. = 4.78) and 3.15 years as managers (*S*.*D*. = 3.17), whereas males had 15.31 years of working experience (*S*.*D*. = 5.60) and 8.38 years as manager (*S*.*D*. = 5.33). In terms of traditional foreign assignments, 69.7% of the respondents presented no foreign working experience, with no differences between females and males. The firms where they had been working were mainly small and medium enterprises (56% of the respondents worked in companies with less than 250 employees), primarily in the manufacturing industry. A total of 71.7% of the respondents were married and, on average, had 10 years of marriage (*S*.*D*. = 5.9); a total of 43.4% of them did not have children.

### Dependent variables

#### Boundaryless career

Boundaryless career was measured considering three types of physical mobility dimensions, namely crossing organizational, industrial, and geographical boundaries. Prior research has mainly reduced the operationalization of a physical boundaryless career to inter-organizational mobility (Sullivan, 1999; Inkson et al., 2012; Gubler et al., 2014a). We add to the ongoing debate that draws attention to the need of developing measures that better capture the original construct, introduced by Sullivan and Arthur (2006), who also included—within the concept of physical mobility—industrial and geographical domains. Similarly, Bidwell and Briscoe (2010) suggest the relevance of geographical mobility as a further dimension of the boundaryless career, and a recent empirical research confirmed that the preference for moving across geographical boundaries can be considered part of the boundaryless career construct (Gubler et al., 2014a). Therefore, with the aim of investigating in depth the “boundaryless career space” (Gubler et al., 2014a), we proposed adding two more dimensions to the traditional organizational mobility. First, we included the movements of the managers across different industrial boundaries. Second, we considered changes in different geographical locations. Specifically, we asked the managers of the sample to indicate: (a) organizational mobility: the number of firms they worked for; (b) industry mobility: the number of different sectors they operated in; and (c) geographical mobility: the number of times they worked abroad for at least 1 year, all with reference to the period of time from T1 to T2. We measured geographical mobility in terms of time spent in a foreign country for a period longer than 1 year instead of in terms of time spent in different regions of the same country. This decision is motivated by the fact that in Italy and in Europe in general the mobility within the same country is not perceived as a form of boundary crossing, due to the limited geographical distances and the same cultural background that characterize the regions of the same country.

We divided the three indicators by the number of years since the end of the MBA to obtain comparable data among managers who finished their course in different years, and consequently may have had different opportunities to practice their ESCs and pursue a boundaryless career and higher objective career success. Then, we included these three measures in the PLS-path modeling to operationalize the variable “boundaryless career.”

#### Objective career success

Objective career success was assessed by using the number of promotions earned by each manager from T1 to T2 that is consistent with a dynamic concept of career as a process of individual change over time. We conceptualized promotions as “any increases in level and/or any significant increases in job responsibilities or job scope” (Seibert et al., 2001, p. 227). Thus, this definition enables us to consider not only vertical progressions but also horizontal career transitions. We weighted the number of promotions with respect to the different number of years after the MBA, in order to take into account that the different time lag may impact on the opportunity to earn promotions. Because the data on promotions was skewed (skew = 1.45, test *p* < 0.01; D'Agostino and Pearson, 1973) we transformed this variable using a natural logarithmic transformation. Such transformation is consistent with the practice of other studies on objective career success (Judge et al., 1995; Seibert et al., 2001).

### Independent variables

Unlike prior studies that usually assess competencies through self-reported measures, we adopted behavioral measures of competencies as rated by the research team to evaluate managers' ESCs (Amdurer et al., 2014). In so doing we mitigated the issue of social desirability (Paulhus and Reid, 1991) and other possible biases and unreliable responses associated with self-assessment (Dunning et al., 2004). At the end of each MBA edition, we administrated a Behavioral Event Interview (Boyatzis, 1998; McClelland, 1998) to its participants. This interview is a development of the Critical Incident Interview technique (Flanagan, 1954), where the attention of the researcher is focused on gathering information on recent (last 12 months) and specific events/situations with definite effects in which the interviewee felt effective or ineffective, and—consequently—not on his/her general opinions or evaluations.

This kind of interview, like the storytelling technique (Martin, 1982; Boje, 1991), has been widely used to structure qualitative data analysis in order to obtain rich and detailed information on the context, behaviors, and strategies adopted to achieve particular goals (Chell, 2004; Campion et al., 2011). Therefore, the Behavioral Event Interview provides a more accurate recording of the person's action, internal thoughts and consequently the underlying intent, and—as demonstrated by prior studies—it has shown a higher predictive validity than respondent measures (Boyatzis, 2009; Ryan et al., 2009). Each interview lasted on average 2 hours and contained the description of five events that had occurred during the previous year. The interview protocol required the collection of three positive and two negative incidents. Once the manager recalled an event, he or she was guided through telling the story of the event with a set of questions, such as: What led up to the situation?, Who said or did what to whom?, What did you say or do next?, What were your thoughts and feelings?, What was the result of the event? (Boyatzis, 2009). In summary, we asked probing and follow-up questions to attain, for each episode, more information on the situation, thoughts, feelings, dialogues, behaviors, and outcomes.

Interviews were recorded, transcribed and coded using a thematic analysis process (Boyatzis, 1998) according to an adapted version of Boyatzis' (Boyatzis, 1982; Boyatzis et al., 1995) and Spencer and Spencer's (1993) codebook, which included seven emotional competencies (Efficiency Orientation, Achievement Orientation, Planning, Initiative, Consciousness, Self-control, Flexibility) and 12 social competencies (Empathy, Persuasiveness, Networking, Negotiating, Self-confidence, Group Management, Developing Others, Oral Communication, Service Orientation, Organizational Awareness, Teamwork, Leadership). Table 1 reports the description of the aforementioned competencies. The interviews were coded by two trained coders and the inter-rater reliability estimates showed a high level of agreement (Cohen's κ = 0.87).

**Table 1:** **Emotional and social competencies included in the codebook**.

**EMOTIONAL COMPETENCIES**
*Achievement orientation:* capacity to ask yourself for high quality standards to try to constantly improve your results, setting challenging and measurable goals, calculating risks and acquiring and transmitting continuously new ways to improve.
*Efficiency orientation*: capacity to perceive input/output relationships and includes the concern for increasing the efficiency of action.
*Planning*: capacity to identify and organize future, or intended actions with a result or direction.
*Initiative*: capacity to take action to accomplish something, and to take this action prior to being asked or forced or provoked into it.
*Conscientiousness*: capacity to seek order and predictability by reducing uncertainty.
*Flexibility:* capacity to adapt to changing circumstances, or alter one's behavior to better fit the situation.
*Self-control*: capacity to inhibit personal needs, or desires for the benefit of organizational, family, or group needs.
**SOCIAL COMPETENCIES**
*Empathy*: capacity to sense others' feelings and perspectives and taking an active interest in their concerns.
*Persuasiveness*: capacity to convince another person, or persons of the merits of, or to adopt, an attitude, opinion, or position.
*Networking*: capacity to build relationships, whether they are one-to-one relations, a coalition, an alliance, or a complex set of relationships among a group of people.
*Negotiating*: capacity to stimulate individuals or groups toward resolution of a conflict.
*Self*-*confidence*: capacity to consistently display decisiveness or presence.
*Group Management*: capacity to stimulate members of a group to work together effectively.
*Developing Others*: capacity to stimulate someone to develop his/her abilities or improve their performance toward an objective.
*Oral Communication*: capacity to explain, describe, or tell something to others through a personal presentation.
*Service Orientation:* capacity to focus mainly on the satisfaction that you provide to others, showing to be helpful and adapting your services or products to their needs.
*Organizational Awareness*: capacity to locate and decipher social networks and power relations, being able to understand the “political” balance in any organization and guiding values and unspoken rules that govern the behavior of its members.
*Teamwork*: capacity to be respectful, collaborative and available to the group, inducing others to engage actively and enthusiastically in the common cause, reinforcing the team spirit and encouraging the participation of all.
*Leadership*: capacity to lead others and triggering phenomena involving emotional resonance, instilling a sense of pride and inspiring people through a compelling vision, bringing out their best aspects.

*Source: adapted from Boyatzis (1982), Spencer and Spencer (1993), and Boyatzis et al. (2015)*.

According to the above codebook, each competency may be described and recognized through a set of several independent behavioral indicators, whose number depends on each competency and may vary from 2 to 6. The coding process identified, for each of the five episodes described in an interview and for each of the 19 competencies we analyzed, how many behaviors performed by the interviewee could be associated to the behavioral indicators present in the codebook. In other words, for each of the interviewees and for each of the 19 analyzed competencies we obtained a matrix with five rows (the number of described episodes) and *i* columns corresponding to the behavioral indicators associated to that competency. The values inside the matrix are the number of times a specific behavior performed by the interviewee is associated to the corresponding behavioral indicator (for each described episode). Consequently, we captured two dimensions: (a) the number of times a competency is expressed through the activation of the same behavioral indicator (frequency), and (b) the number of different behaviors associated with the same competency adopted by the interviewee (variety). Since an interviewee may demonstrate the possession of a competency through the activation of few or many indicators, and may use them a few times or recurrently, we built an index to capture the overall intensity of the possession of a competency by considering both the frequency and variety dimensions.

Let *n*_*m*_ be the number of episodes described by the *m*-th manager and let *i*_*c*_ be the number of indicators associated to the *c*-th competency. We defined as *I*_*c, e, m*_ the number of indicators of the *c*-th competency activated in the *e*-th episode by the *m*-th interviewee. Then we considered the following variable, which gives a measure of the variety of indicators used in the *e*-th episode by the *m*-th interviewee for a *c*-th competency and rewards those who used more than a half of them:

(1) $$v_{c,e,m}=\frac{I_{c,e,m}}{\max\left(I_{c,e,m},\left(i_{c}-I_{c,e,m}\right)\right)}$$

Similarly, we defined as *E*_*c, i, m*_ the number of episodes in which the *i*-th indicator of the *c*-th competency has been activated by the *m*-th manager. Then we considered the following variable, which gives a measure of the frequency with which the *i*-th indicator of the *c*-th competency has been used by the *m*-th interviewee, and rewards those who used that indicator in more than a half of the episodes:

(2) $$f_{c,i,m}=\frac{E_{c,i,m}}{\text{max}\left(E_{c,i,m},\left(n_{m}-E_{c,i,m}\right)\right)}$$

Finally, we computed the index for the intensity of each single competency included in the model with the following formula:

(3) $$CI_{c,m}=log\left(1+\frac{F_{c,m}V_{c,m}}{N}100\right)$$

where *N* = (1 + number of episodes without activated indicators for the *m*-th manager) ^*^ (1 + number of indicators of the *c*-th competency never utilized in the episodes considered). *F*_*c, m*_ and *V*_*c, m*_ are, respectively, the mean of *f*_*c, i, m*_ and *v*_*c, e, m*_.

(4) $$F_{c,m}=\frac{1}{n_{m}}\sum_{i\;=\;1}^{n_{m}}f_{c,i,m}\text{ and }V_{c,m}=\frac{1}{i_{c}}\sum_{e\;=\;1}^{i_{c}}v_{c,e,m}$$

The Competency Index *CI* computed for each emotional and social competency is designed specifically to take into account both the variety of behavioral indicators activated within a given competency, and how frequently they are used (systematically or occasionally) among the episodes told by a manager. The higher the value of the index, the higher the ability of the manager to manifest different competencies. The Appendix in Supplementary Material provides some examples of the construction of the index.

In the PLS-path modeling that we used to test the relationships among our variables, the two independent variables regarding the emotional and social competencies will be obtained by a linear combination of the Competency Indexes *CI* of each competency that belongs to its corresponding cluster. The weight of the combination will be estimated by PLS-path modeling.

### Controls

Prior research found that variables such as gender and working experience affect physical career mobility and career success (Judge et al., 1995; Nabi, 1999; Valcour and Tolbert, 2003; Segers et al., 2008). In our study, gender was measured as a dichotomous variable (dummy coding 0 male, 1 female). Work experience was directly measured in terms of number of years. Moreover, we controlled for the different educational background of the managers, distinguishing between those with an engineering degree and managers with an economic/law degree. Specific disciplinary backgrounds may increase the opportunities to undertake career mobility and to obtain promotions.

## Findings

Descriptive statistics (from Time 1 concerning the ESCs and from Time 2 the remaining variables) and two-way correlations for the constructs used in the analysis are shown in Table 2.

**Table 2:** **Means, standard deviations and correlations**.

	**Variables**	**Mean**	***S.D*.**	**1**	**2**	**3**	**4**	**5**	**6**
1	Efficiency orientation	1.383	0.933						
2	Planning	0.731	0.685	0.360^***^					
3	Initiative	0.857	0.645	0.210^*^	0.030				
4	Consciousness	1.866	1.194	0.186^*^	0.303^***^	0.059			
5	Self-control	0.498	0.748	−0.115	0.046	0.008	0.191^*^		
6	Flexibility	0.743	0.904	−0.033	0.040	−0.030	0.063	0.265^**^	
7	Achievement orientation	1.360	0.879	0.137	0.120	0.192^*^	0.107	0.028	0.103
8	Empathy	0.875	0.828	−0.008	0.090	0.141	0.319^***^	0.280^***^	0.185^*^
9	Persuasiveness	0.250	0.323	0.078	0.210^*^	0.093	0.298^***^	0.118	0.008
10	Networking	0.536	0.761	−0.046	−0.070	0.070	0.023	0.002	0.071
11	Self-confidence	1.496	0.938	0.078	0.058	0.289^***^	0.090	0.237^**^	0.105
12	Group management	0.145	0.320	0.059	0.142	0.044	0.034	0.024	−0.024
13	Developing others	0.186	0.377	0.117	0.112	0.082	0.009	0.105	0.338^***^
14	Oral communication	0.167	0.240	0.090	0.142	0.317^***^	0.321^***^	0.166^*^	0.129
15	Service orientation	0.373	0.557	0.010	−0.042	0.153	0.057	0.125	0.169^*^
16	Negotiating	0.173	0.344	−0.027	−0.077	−0.088	0.157	−0.025	−0.085
17	Organizational awareness	0.228	0.438	0.017	0.062	0.226^**^	0.128	0.151	0.110
18	Teamwork	0.964	0.858	0.190^*^	0.146	0.141	0.356^***^	0.276^***^	0.124
19	Leadership	0.346	0.572	0.217^**^	0.244^**^	0.158	0.163	0.037	−0.086
20	Work Experience	14.613	5.812	0.007	0.058	−0.025	−0.271^**^	−0.227^**^	−0.074
21	Gender	0.859	0.349	0.048	−0.008	0.143	−0.240^**^	−0.225^**^	−0.138
22	Disciplinary background	1.585	0.495	−0.002	−0.096	−0.108	−0.058	−0.106	0.010
23	Organizational mobility	0.422	0.538	0.088	−0.014	0.018	0.315^***^	0.162	0.377^***^
24	Industry mobility	0.725	1.007	0.077	0.068	−0.017	0.330^***^	0.269^**^	0.424^***^
25	Geographical mobility	0.123	0.300	0.091	0.088	−0.086	0.225^**^	0.052	0.164
26	Career success	0.309	0.347	−0.008	0.035	−0.002	0.110	0.055	0.041
	**Variables**	**7**	**8**	**9**	**10**	**11**	**12**	**13**	
8	Empathy	−0.021							
9	Persuasiveness	0.099	0.313^***^						
10	Networking	−0.098	0.117	0.076					
11	Self-confidence	0.058	0.264^**^	0.138	0.173^*^				
12	Group management	0.152	0.039	0.155	−0.107	−0.118			
13	Developing others	0.116	0.214^*^	0.075	−0.018	0.116	0.219^**^		
14	Oral communication	0.130	0.315^***^	0.116	0.040	0.198^*^	−0.031	0.101	
15	Service orientation	0.137	0.038	0.138	0.142	0.052	0.015	−0.013	
16	Negotiating	−0.104	0.088	0.200^*^	−0.019	−0.018	−0.097	−0.196^*^	
17	Organizational awareness	0.101	0.222^**^	0.250^**^	−0.014	0.006	0.099	0.059	
18	Teamwork	0.131	0.143	0.212^*^	−0.041	0.039	0.246^**^	0.107	
19	Leadership	0.232^**^	0.241^**^	0.208^*^	−0.074	0.148	0.387^***^	0.190^*^	
20	Work Experience	−0.059	0.124	0.039	0.073	−0.029	0.035	0.189^*^	
21	Gender	−0.118	−0.025	0.077	0.003	−0.005	−0.058	0.004	
22	Disciplinary background	−0.044	−0.044	−0.060	0.194^*^	−0.077	−0.147	−0.195^*^	
23	Organizational mobility	0.001	0.249^**^	0.071	0.175^*^	0.031	−0.088	0.027	
24	Industry mobility	0.080	0.254^**^	0.084	0.128	0.099	−0.076	0.219^**^	
25	Geographical mobility	0.065	0.077	0.119	0.084	−0.009	−0.011	0.177^*^	
26	Career success	0.018	0.099	−0.028	0.176^*^	0.151	0.099	0.229^**^	
	**Variables**	**14**	**15**	**16**	**17**	**18**	**19**	**20**	
15	Service orientation	0.062							
16	Negotiating	0.042	−0.037						
17	Organizational awareness	0.097	0.052	−0.053					
18	Teamwork	0.098	0.203^*^	−0.066	0.094				
19	Leadership	0.236^**^	0.066	−0.101	0.013	0.274^***^			
20	Work Experience	0.082	−0.059	−0.108	−0.047	−0.376^***^	0.143		
21	Gender	−0.092	−0.067	0.042	−0.055	−0.219^**^	0.120	0.322^***^	
22	Disciplinary background	−0.139	−0.109	0.162	−0.070	−0.251^**^	−0.241^**^	0.104	
23	Organizational mobility	0.236^**^	0.067	0.055	0.240^**^	0.268^**^	−0.091	−0.192^*^	
24	Industry mobility	0.293^***^	0.076	0.072	0.250^**^	0.177^*^	−0.039	−0.051	
25	Geographical mobility	0.176^*^	0.015	0.118	0.307^***^	0.136	0.122	−0.025	
26	Career success	0.100	−0.015	−0.078	−0.015	0.010	0.090	−0.100	
	**Variables**	**21**	**22**	**23**	**24**	**25**			
22	Disciplinary background	−0.054							
23	Organizational mobility	−0.222^**^	0.076						
24	Industry mobility	−0.035	0.006	0.767^***^					
25	Geographical mobility	−0.043	−0.052	0.310^***^	0.413^***^				
26	Career success	−0.011	−0.037	0.231^**^	0.259^**^	0.184^*^			

* p < 0.05;

** p < 0.01;

*** *p < 0.001 (two tailed tests)*.

**Figure 1:**
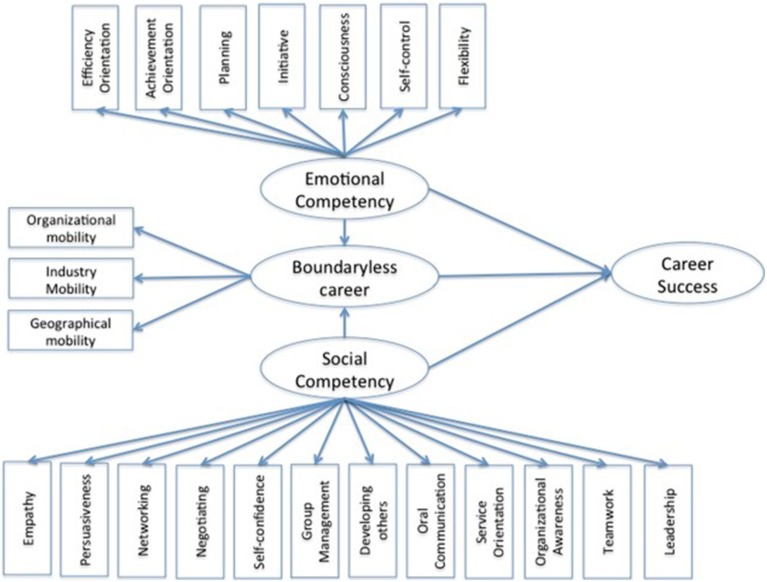
**Conceptual model**.

To test our hypotheses on the impact of ESCs on physical boundaryless careers and objective career success we carried out a partial least squared-path modeling (PLS-PM) using R software and in particular the package plspm (Sanchez, 2013). Figure 1 depicts our conceptual model. The PLS-PM can be viewed as a technique that allows for the analysis of a system of linear relationships between blocks of several variables and it is also a useful method for prediction purposes. Moreover, this approach is distribution-free, that is it requires no assumptions on statistical distribution of the measurable and latent variables.

The PLS-PM encompasses two models: the measurement model, or outer model, and the structural model, or inner model. In the measurement model the variables form blocks that measure the latent variables. These blocks of variables are formative or reflective in type. In our model the blocks are of reflective type and the latent variables are *boundaryless career, emotional competencies*, and *social competencies*. The inner model represents the hypothesized relationships in the structural equations. In our case the inner model is composed of two equations. The first one considers the relationship among *emotional* and *social competencies*, on the one side, and *boundaryless career* on the other side. The second one expresses the relationship among *emotional* and *social competencies, boundaryless career*, and *career success*.

With regards to the reflective measures, we assessed the following characteristics: unidimensionality of the indicators, convergent and discriminant validity.

For the unidimensionality of the indicators, we considered the Composite Reliability Index (Wertz et al., 1974), which was above the recommended threshold of 0.7 for *boundaryless career* (0.86), and for the *emotional competencies* (0.70), whereas for *social competencies* (0.68) it was only slightly below the threshold, so these results sustain the unidimensionality of the constructs. This index is considered to be a better indicator than the Cronbach's α because it does not assume the so-called *tau* equivalence of the manifested variables and takes into account to what extent the latent variable explains its block of indicators (Chin, 1998).

Regarding the second aspect, that is convergent and discriminant validity, as highlighted in Table 3, some of the *emotional* and *social competencies* were considered appropriate to be excluded from the analysis because their values were below the threshold that we fixed, considering our sample size, at 0.50 in accordance with the recommended threshold (Hair et al., 1998). More precisely, for the *emotional competencies* we kept Consciousness, Self-control, and Flexibility, whereas for the *social competencies* we kept Empathy, Networking, Self-confidence, and Developing Others. Table 3 reports the loadings of the measurable variables considering, on the one hand, all the original ESCs and, on the other hand, only the competencies included in the final PLS-PM. Finally, the cross-loadings reported in Table 4 confirm the discriminant validity of the constructs.

**Table 3:** **Loadings for the complete and reduced model yielded by PLS-PM model**.

**Competency**	**Construct**	**Complete**		**Reduced**	
		**Weights**	**Loadings**	**Weights**	**Loadings**
Efficiency orientation	Emotional	0.1152	0.2272		
Planning	Emotional	0.1012	0.3697		
Initiative	Emotional	0.0214	0.1105		
Consciousness	Emotional	0.6140	0.7523	0.6545	0.7364
Self-control	Emotional	0.2686	0.4924	0.2880	0.5349
Flexibility	Emotional	0.5189	0.6183	0.5558	0.6549
Achievement orientation	Emotional	0.0811	0.2349		
Empathy	Social	0.2792	0.6630	0.4378	0.6570
Persuasiveness	Social	0.0619	0.4064		
Networking	Social	0.3352	0.4092	0.5238	0.6294
Self-confidence	Social	0.1839	0.4398	0.2878	0.5424
Group management	Social	0.0307	0.1476		
Developing others	Social	0.2758	0.4390	0.4240	0.5345
Oral communication	Social	0.3515	0.5852		
Service orientation	Social	0.0528	0.2119		
Negotiating	Social	0.0255	−0.0229		
Organizational awareness	Social	0.2821	0.4387		
Teamwork	Social	0.2363	0.4013		
Leadership	Social	0.0340	0.3299		
Organizational mobility	Boundaryless	0.4878	0.9077	0.5011	0.9152
Industry mobility	Boundaryless	0.4278	0.9318	0.4296	0.9336
Geographical mobility	Boundaryless	0.2604	0.6091	0.2374	0.5911

**Table 4:** **Crossloadings**.

**Competency**	**Construct**		
	**Emotional competencies**	**Social competencies**	**Boundaryless career**
Consciousness	0.6831	0.1859	0.3560
Self-control	0.5997	0.2465	0.2138
Flexibility	0.6983	0.3121	0.4163
Empathy	0.3857	0.6895	0.2560
Networking	0.0551	0.5324	0.1638
Self-confidence	0.1922	0.5265	0.0584
Developing others	0.2343	0.6164	0.1520
Organizational mobility	0.4538	0.2135	0.9137
Industry mobility	0.5255	0.3086	0.9265
Geographical mobility	0.2426	0.1574	0.5643

Before testing for significance of path coefficients in the model, we tested for collinearity in the independent and control variables. In doing so, we considered the Variance Inflation Factor (VIF), whose results are summarized in Table 5. All the variables present a VIF-value below the threshold of 5, as recommended (Hair et al., 2013).

**Table 5:** **Variance inflation factor**.

**Variables**	**Variance inflation factor**
Emotional competencies	1.6039
Social competencies	1.2836
Boundaryless career	3.3238
Work experience	3.0088
Gender	1.0499
Disciplinary background	1.3434

**Figure 2:**
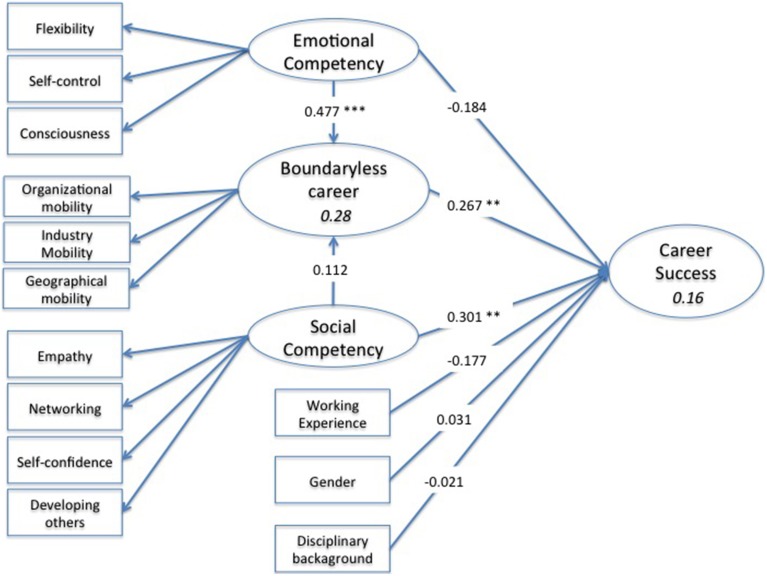
**Reduced estimated model**. ^*^ *p* < 0.05; ^**^ *p* < 0.01; ^***^ *p* < 0.001.

The results, reported in Table 6, show that *emotional competencies* positively predict physical career mobility. Furthermore, findings highlight that *social competencies* and *boundaryless career* have a positive impact on *career success*. The control variables are not significant. The final model is depicted in Figure 2.

**Table 6:** **Path coefficients**.

	**Estimate**	***p*-Values**
**BOUNDARYLESS**		
Intercept	0.000	1.000
Emotional	0.477	0.000
Social	0.112	0.178
Work Experience	−0.026	0.754
Gender	0.015	0.851
Disciplinary background	0.069	0.347
**SUCCESS**		
Intercept	−0.000	1.000
Emotional	−0.184	0.079
Social	0.301	0.001
Boundaryless	0.267	0.005
Work Experience	−0.177	0.051
Gender	0.031	0.723
Disciplinary background	−0.021	0.794

The coefficients of determination (*R*^2^)-value for each of the two structural equations are shown in Table 6. These indexes are 0.28 for the *boundaryless career* equation and 0.16 for the *career success* equation. The bootstrap test of significance led us to reject the null hypothesis of *R*^2^ = 0, so the equations turned out to be significant for the purpose of this study.

To study the possible indirect effect of *emotional* and *social competencies* on *career success* through *boundaryless career*, we empirically tested a mediating model performing the Aroian (1947) version of the Sobel test suggested by Baron and Kenny (1986)^1^. The results (Table 7) highlight the full mediation effect on the relationship between *emotional competencies* and *career success*, whereas no mediation effects are detected for *social competencies*.

**Table 7:** **Mediation model**.

	**Aroian**		**Mediation**
	**Statistics**	***p*-values**	
EC -> Boundaryless -> Success	2.504	0.012	Full
SC -> Boundaryless -> Success	1.223	0.221	No

## Discussion

Our results show the presence of some significant relationships between ESCs, a boundaryless career and career success. First, we focus our attention on the antecedents of a physical boundaryless career. As expected, we found that emotional competencies have an impact on the physical career mobility. *Flexibility, self-control*, and *consciousness* are the competencies that contribute, in our model, to build the emotional competencies construct, which in turn shows a significant relation with the variable *boundaryless career*. This means that the adoption of career paths that cause managers to change their environment in terms of different firms, industries or countries, is supported by—and at the same time contributes to enhance (Zhu et al., 2013)—some personal capabilities that place them in the position of being able to easily modify their behaviors, to adapt themselves quickly to unexpected changes (or even to react properly when an expected change doesn't happen), and being able to manage the stressors coming from the need for adaptation to new conditions (O'Connell et al., 2008). Emotional *self-control* makes it possible to not only be able to better face stressful situations but also to avoid the sacrifice syndrome (Boyatzis and McKee, 2005) typical of managerial positions, improving the overall managerial performance (Kuijpers et al., 2006; de Boer et al., 2015). In addition, the competency *consciousness* (that is the capability to meet one's own commitments and be accurate in performing a task) helps the managers to continue to maintain a strong focus on the qualitative level of their results and on the details of their actions, without reducing their performance, even in different environments (Rajadhyaksha, 2005). Consequently, considering the variables included in our model, H1 is supported.

On the other hand, in our sample the hypothesized relationship between social competencies and a boundaryless career does not find a confirmation (H2 not supported). This result seems to reinforce some arguments present in the literature, which underline that “people who see their career as unbounded to their present organization might be less inclined to invest in their relationships at work” (Verbruggen, 2012, p. 289). Consequently, it would appear that investments in relational capital do not help them to expand their career beyond the traditional boundaries.

Second, our results show which are the determinants of objective career success. Interestingly, emotional competencies are not directly related to career advancements (H3 is not supported). Consequently, even if emotional competencies do impact on work performance (it is worth remembering that in our sample emotional competencies have been drawn from the analysis of the behavioral event interviews, which focused on specific results of effectiveness experienced by the manager), at the same time they may not be enough to directly favor higher professional advancements in terms of hierarchical progressions and promotions. Specifically, our findings underline the relationship between career success and social competencies. In particular, *empathy, networking, self-confidence*, and *developing others* are the competencies which contribute, in our model, to build the social competencies construct, which in turn shows a significant relation with objective career success (H4 is supported). This result is consistent with some previous studies on the impact of social competencies on job advancement (Mak et al., 1994; Atwater and Pittman, 2006; Wolf, 2010) but, in addition, it reinforces the specific role of the capability to understand others and others' feelings, paying attention to others' needs and being sincerely interested in others' problems. This kind of constant attention creates a better organizational climate reducing conflicts (Palanski, 2012), supports a process of organizational healing (Powley, 2013), improves the social acceptance of those who express it and—consequently—increases an individual's endorsement and makes him/her eligible for more advanced positions. However, another component of managers' career success is made up of their capability to build networks, which helps them to create a relational environment that facilitates the development of trust and access to career opportunities (Rasdi et al., 2013). By building networks, managers can exploit new knowledge domains to solve problems, create new opportunities for their firms, improve their internal and external reputation, access information useful for their decisions and consequently improve their performance (Orpen, 1996; de Janasz and Forret, 2008; Wolff and Moser, 2009). In addition, the capability to develop colleagues and subordinates, which is typical of the coaching leadership style (Goleman et al., 2002), supports the improvement of the team's performance, satisfaction and results, generates a good organizational climate (Kim et al., 2013) and a sense of recognition that can impact on organizational outcomes and consequently favor some opportunities of career advancement. Likewise, the capability of the managers to present themselves as self-confident creates a positive and proactive approach to problems (McCarthy, 2002), helps them to take the risks needed to persevere in working toward their visions (Shipman and Mumford, 2011), increases the followers' commitment (Luthans and Peterson, 2002) and helps to seek different alternative ways to make things happen, by generating assumptions for subsequent success. Progression in the career path implies new tasks in terms of people management activities, a careful attention to the organizational climate, the creation of relations of trust and a lower degree of involvement in operative activities. Thus, employees who possess a higher level of social competencies may be assessed as the best candidates for higher managerial positions (Seibert et al., 2001).

In addition to the social competencies, objective career success is related, in our results, to the adoption of a boundaryless career (H5 is supported). As a consequence, since emotional competencies are not directly related to career advancement, the relation between emotional competencies and objective career appears to be fully mediated by the adoption of career choices that go beyond the traditional boundaries. Therefore, the choice of a boundaryless career represents an opportunity in terms of the experience it provides to the manager and—showing how he/she has been able to successfully manage changing situations and environments—in terms of the signal it gives to the employer, which both lead to career advancements.

Third, the control variables do not play any significant role in the analyzed relationships. Working experience, gender and educational background seem not to impact on the relation between ESCs and career outcomes, which are mainly influenced by the individual behaviors and career choices of the managers. This reinforces our analysis in terms of the relevance of the ESCs to accomplish a higher career success.

Finally, by considering the overall relationship between competencies and objective career success it is interesting to note that some groups of competencies are involved in building consistent constructs. This result confirms the presence of combinations, or configurations, of competencies whose relation generates a higher performance (Camuffo et al., 2009) and has to be taken into consideration for developmental purposes, since it suggests combining different behaviors to obtain better results.

## Research contributions, implications, and limitations

This study contributes to the literature in several ways. Firstly, adding to the boundaryless career research, this study sheds new light on the antecedents and outcomes related to an individual's physical career mobility. It advances knowledge on the relevance of emotional competencies in enabling managers to face the ambiguity and uncertainty usually associated with a move toward a different organization, sector and geographical location. This paper also provides empirical evidence on the positive relationship between physical mobility and objective career success. As recently highlighted in the literature, an in-depth understanding of the characteristics of contemporary career paths requires a theoretical advancement in their determinants and outcomes (Rodrigues and Guest, 2014). Our study provides initial insights into this issue by opening promising lines of research that can further examine the role of behaviors in explaining the propensity to pursue a cross-boundary career, beyond the current research that mainly focuses on individual attributes (gender, age, prior experience). Moreover, we have contributed to the debate on the operationalization of the boundaryless construct, and specifically on its physical dimension. We considered different facets of the concept, not only looking at inter-organizational changes but also including mobility across industries and countries. In order to advance the conceptualization of the “boundaryless career space” future studies should include further dimensions of physical mobility, such as functional or professional changes.

Secondly, the paper's contribution lies in the research field of emotional and social competencies. Not only are ESCs confirmed to be relevant predictors of job performance, as extensively demonstrated by the literature, but we found that they play a salient role in influencing individual career decisions and subsequent success. Our study also adds to the operationalization of the emotional and social competencies constructs, introducing the Competency Index that captures both the frequency and variety dimensions. To date, prior research has only considered the first of these two dimensions (Amdurer et al., 2014). Moreover, using a behavioral measure of competencies, through the administration of Behavioral Event Interviews, we also mitigate the limits of self-report and single-respondent bias that characterize those studies that rely on self-assessment of emotional and social competencies.

Finally, data collection on independent and dependent variables were not carried out through the same survey but we gathered data at different points in time. Thus, unlike prior boundaryless career and competency-based research that adopts a cross-sectional design, our study represents the first attempt to provide evidence of a long-term impact by the possession of ESCs on subsequent physical mobility and career promotions. Future studies may continue to develop a longitudinal research design in order to extend our findings, considering further dimensions of career mobility and outcomes.

These findings also present some managerial implications. A first implication is for the educational institutions, which could design training programs that aim to develop ESCs and monitor over time their development, as well as their participants' subsequent career paths and advancement. Specifically, our findings underline a different effect of emotional and social competencies on the individual career paths and provide a better understanding of those specific ESCs that have a higher impact in favoring boundaryless career and promotion advancements. Even if the benefits of career mobility depend on individual career and life goals, some emotional competencies found in this study may help individuals to face the potentially stressful nature of changing jobs. This could have significant implications in terms of investing in those specific sets of competencies in terms of management education and training. Moreover, companies may modify their selection techniques and redesign their training processes and career plans by considering those specific ESCs that impact mostly on career mobility and objective career success. With particular regards to boundaryless careers, companies that promote mobility across organizational boundaries and international careers that need to be able to identify candidates for expatriate positions may benefit from the assessment of the emotional competencies of their employees. Regarding objective career success, firms may consider evaluating in their career management practices the assessment of employees' social competencies and prior experiences in different organizational, industrial and geographical contexts.

Some limitations and recommended directions for future research can be identified.

Future research should replicate the findings with larger samples that are more heterogeneous in terms of geographical, cultural, and gender composition. With specific regard to the gender variable, despite the fact that our research shows that there are no differences between men and women in career mobility decision and success and in their demonstration of ESCs, we were not able to provide further support for this result due to the limited number of females in our sample, which is consistent with the wider gender composition of the managerial class in Italy. Indeed, gender may impact on career paths because of a variety of work-life factors, such as different mobility propensity between men and women and diverse motivational drivers (promotions in the same company vs. interfirm promotions), etc.

In our research, we also considered the temporal dimension so as not to penalize those managers who presented a shorter length of working experience. Indeed, we applied a corrective factor in measuring boundaryless career and objective career success, but in so doing we hypothesized that career paths may be linear with regards to time. Further studies should consider alternative ways to include the temporal variables. The specific economic situation that individuals may face at certain periods of time can influence their own perceptions in terms of career opportunities but can also affect the career advancements that companies provide them.

### Conflict of interest statement

The authors declare that the research was conducted in the absence of any commercial or financial relationships that could be construed as a potential conflict of interest.

## Footnotes

^1^Let *a* be the regression coefficient for the association between independent variable and mediator and σ_*a*_ its standard error. Similarly the coefficient for the association between the mediator and the dependent variable has been denoted by *b* (when the independent variable is also a predictor of the dependent variable) and σ_*b*_ is the standard error of *b*. The Sobel statistic $\frac{ab}{\sqrt{b^{2}\sigma_{a}^{2}\;+\;a^{2}\sigma_{b}^{2}}}$ and the Aroian statistics $\frac{ab}{\sqrt{b^{2}\sigma_{a}^{2}\;+\;a^{2}\sigma_{b}^{2}\;+\;\sigma_{a}^{2}\sigma_{b}^{2}}}$ are very similar, but the first omits a term of the variance estimate in the denominator, therefore the Aroian statistics avoid making the assumption that the product of σ_*a*_ and σ_*b*_ is vanishingly small.
