# Supplementary material for: Boundaryless career and career success: the impact of emotional and social competencies
Source: Front Psychol. 2015 Sep 1;6:1304. doi: 10.3389/fpsyg.2015.01304 (PMC4554953; doi:10.3389/fpsyg.2015.01304)
Supplement: Supplementary file 1 [file Appendix.PDF]

APPENDIX

The appendix provides four examples of the calculation of the index introduced in section 3.3 with the aims, on one hand, to clarify the construction procedure and, on the other hand, to point out its capacity to measure consistently different response patterns recorded by BEIs. All the examples are supposed to measure the competency by means of five episodes *E* and four behavioral indicators *B*, thus the data is organized in a matrix 5x4. Starting from this matrix we compute:

- using formula (1), $I_{c,e,m}$ and $v_{c,e,m}$ (reported on the right of the data matrix),
- using formula (2), $E_{c,i,m}$ and $f_{c,i,m}$ (reported below the data matrix),
- using formula (4) $V_{c,m}$ and $F_{c,m}$ (on the bottom right of the table);
- using formula (3) the Competency Index *CI*

Example *A:* the interviewee activates all the behavioral indicators in only one episode.

	B1	B2	B3	B4	Total
E1	1	1	1	1	4
E2	0	0	0	0	0
E3	0	0	0	0	0
E4	0	0	0	0	0
E5	0	0	0	0	0
Total	1	1	1	1	

$I_{c,e,m}$	$v_{c,e,m}$
4	1
0	0
0	0
0	0
0	0

episodes with no indicators 4

$E_{c,i,m}$	1	1	1	1
$f_{c,i,m}$	0.25	0.25	0.25	0.25

indicators never activated 0

$V_{c,m}$	0.200
$F_{c,m}$	0.250
N	5.000
CI	**0.693**

Example *B*: the interviewee uses only one behavioral indicator in all episodes.

	B1	B2	B3	B4	Total
E1	1	0	0	0	1
E2	1	0	0	0	1
E3	1	0	0	0	1
E4	1	0	0	0	1
E5	1	0	0	0	1
Total	5	0	0	0	

$I_{c,e,m}$	$v_{c,e,m}$
1	0.33
1	0.33
1	0.33
1	0.33
1	0.33

episodes with no indicators 0

$E_{c,i,m}$	5	0	0	0
$f_{c,i,m}$	1	0	0	0

indicators never activated 3

$V_{c,m}$	0.333
$F_{c,m}$	0.250
N	5.000
CI	**1.126**

Example *C*: the interviewee activates several (but not all) indicators in several (but not all) episodes.

	B1	B2	B3	B4	Total
E1	1	0	0	0	1
E2	1	0	0	0	1
E3	0	1	1	0	2
E4	0	1	0	0	1
E5	0	0	0	0	0
Total	2	2	1	0	

$I_{c,e,m}$	$v_{c,e,m}$
1	0.33
1	0.33
2	1.00
1	0.33
0	0.00

episodes with no indicators 1

$E_{c,i,m}$	2	2	1	0
$f_{c,i,m}$	0.67	0.67	0.25	0

indicators never activated 1

$V_{c,m}$	0.400
$F_{c,m}$	0.250
N	5.000
CI	**1.601**

Example D: the interviewee uses all the indicators and he/she uses at least one behavioral indicator in all episodes.

	B1	B2	B3	B4	Total
E1	1	0	0	0	1
E2	0	0	0	1	1
E3	0	1	0	0	1
E4	0	0	1	0	1
E5	0	0	1	0	1
Total	1	1	2	1	

$I_{c,e,m}$	$v_{c,e,m}$
1	0.33
1	0.33
1	0.33
1	0.33
1	0.33

episodes with no indicators 0

$E_{c,i,m}$	1	1	2	1
$f_{c,i,m}$	0.25	0.25	0.67	0.25

indicators never activated 0

$V_{c,m}$	0.333
$F_{c,m}$	0.250
N	5.000
CI	**2.550**
